# The Publishers' Pushback against NIH's Public Access and Scholarly Publishing Sustainability

**DOI:** 10.1371/journal.pbio.1000030

**Published:** 2009-01-27

**Authors:** John Willinsky

## Abstract

Last September, US Congressman John Conyers introduced the Fair Copyright in Research Works Act. John Willinsky explains how the bill's Orwellian title obscures its true aim: to derail the new policy implemented by the National Institutes of Health to open access to publicly funded research, amid talk of sustainability that cuts both ways.

The dying light of the George W. Bush presidency was marked by, among other things, a legislative move to derail recent gains in the federal government's opening of science. In particular, the innocuous sounding “Fair Copyright in Research Works Act” (HR 6845) introduced into the House by John Conyers, Jr. (DEM-MI), on 9 September 2008 [1] was poised to shut down the National Institutes of Health (NIH) Public Access Policy [2], as well as forestall the spread of this open-access spirit to other areas of federally sponsored research and scholarship. Hearings were held, but the bill did not make it through the House. End of story? Not quite.

Certainly, the Obama presidency promises, among so many things, an improved regard for science. Yet I can't help but agree with Peter Suber's prediction that Congress will see the likes of the Fair Copyright in Research Works Act again, though perhaps not from Conyers, who was an early supporter of Obama [3]. The back story on this bill is complicated by the publishers executing an about-face on “archiving rights,” which they have traditionally supported, but it speaks to a larger battle underway that has everything to do with the public standing of research and scholarly work.

Conyers' bill, with strong backing from both the profit and nonprofit scholarly publishing organizations, would preclude federal agencies from retaining any claim on research articles in which the work had been government funded, specifically targeting any right that “involves the availability to the public of that work” [1]. What is held to be “unfair” in the bill is government interference with the publisher's exclusive ownership over research. This is not, however, a case of keeping the government's clumsy hand off a free market. The scholarly publishing market depends on government interference in the first instance. The government allows publishers to exercise monopoly rights over this research through copyright law, a form of market interference warranted by the works' contribution to “the progress of Science and useful Arts,” as the United States Constitution puts it [4]. And if that were not enough, the government also funds directly and indirectly the production, authoring, and reviewing of the content.

What the NIH's Public Access Policy—which had been supported by no less than 33 Nobel Prize laureates, the Taxpayers Alliance for Access, and leading research libraries—had established was a public exception to that monopoly. It was a trade-off that protected the initial intent of the constitutional sense of copyright, as greater access contributes to the progress of science and such useful arts as medicine.

What is strangely amiss in the publishers' support for outlawing the NIH Public Access Policy is that they support the upshot of this initiative with their own current copyright policies. While a growing number of open-access journals provide immediate access to the published version, even subscription-based publishers had originally been happy enough to grant authors the right to do what they were already doing, which was putting PDFs of their articles up on their websites. Still, it was not long before the publishers began to limit authors' archiving rights, imposing an embargo period of perhaps a year following publication after which the author is permitted to archive the final peer-reviewed draft (rather than the published version) [5]. Now, as indicated by their support for the Fair Copyright in Research Works Act, publishers are taking a stand against archiving. As the International Association of STM Publishers recently put it, “publishers do not believe that self-archiving offers a sustainable alternative for scientific publishing” [6].

One reason for the publishers' change of heart is that archiving is catching on, amid growing public expectations that research is a public good that should be made freely available online. Research libraries are setting up dedicated archives for just such purposes. Funding agencies, foundations, and institutions are putting in place policies supporting archiving as a duty and point of pride. Still, when the NIH established its Public Access Policy in April of 2008, the terms carefully reflected the publisher's own archiving policies. Authors were asked to deposit the “final peer-reviewed manuscript” in PubMed Central on publication, which would then be made public no later than 12 months after publication [2]. In this way, the subscription-based publishers had forestalled open access in any strict sense, by ensuring that the public and health professionals had decidedly second-class access to this work.

Yet that wasn't enough to allay the concerns of the publisher associations. Having won archiving restrictions, which were designed to protect the value of journal subscriptions—both for immediacy and accuracy of access—they have decided that archiving policies are a threat in principle. In testifying last September in support of the bill before the Subcommittee on Courts, the Internet, and Intellectual Property Committee on the Judiciary, Martin Frank, Executive Director of the American Physiological Society (APS), insisted that the issue was not access rights but revenue streams [6]. The NIH mandate, he argued, “risks undermining the revenue stream derived principally from subscriptions, that enables publishers to add value to research articles and to enhance readers' ability to discover and use scientists' work.” Publishers report that they are adding as much as US$4,000 value to a research article, while holding that only by virtue of exclusive ownership of this public good can they see their way to an adequate return on investment, in a case of the tail having to own the dog in order to wag it [7].

Frank's stance makes it clear that *access* itself it not the issue. After all, APS's 17 journals already make their contents free after 12 months, contributing to Highwire Press' 2 million freely available articles, exceeding the terms of the NIH mandate (as APS makes the final published version freely available). By the same token, the 73 nonprofit publishers that belong to the DC Principles Coalition, on whose behalf Frank was also speaking, are all about “free access to science,” as its website makes clear (http://www.dcprinciples.org/). What matters, then, as Frank pointed out, was that APS “can modify [its current free access policy] should 12 months prove disadvantageous to the Society's business model” [6]. The point was reinforced by the International STM Publishers Association, who declared in their letter of support for the Fair Copyright bill their concerns regarding embargoed archiving's “potential for harm to scholarly communication” [8]. The association made it clear that its members supported “all business models – all models that ensure continuity and sustainability of the journal model that have brought such significant insight and information to the scientific community.”

For STM publishers, the NIH mandate “puts at risk a system which has enabled more research to be available to more scientists in more countries than at any point in the history of science” [8]. But is the opposite not true? To insist that the current publishing economy must be sustained places the system at risk, and all the more so amid the current economic downturn, which is bound to affect research library budgets in the coming year. The point has been brought home by Heather Joseph, executive director of the Scholarly Publishing and Academic Resources Coalition (SPARC), who testified in opposition to the Fair Copyright bill on behalf of the leading research libraries, which can not sustain subscriptions to as many journals as they would like: “This situation [in which libraries can afford access to only a portion of the literature] is exacerbated by the continued rapid escalation in price of journal subscriptions, which puts libraries in the position of having to cancel subscriptions” [9].

At the root of the problem is the current cost of access to knowledge and how those costs have come to differ so radically from journal to journal that one has to wonder about whether the progress of science is being served. This is the result of the scholarly publishing economy that emerged during the last half of the twentieth century. In that time, the commercial scholarly publishers adeptly served the greatly expanding scale of government-sponsored research by providing many new journal titles in new areas that soon became a critical part of scholarly communication. It was a move that also enabled commercial publishers to run subscription prices up each year well over the rate of inflation, with some nonprofit societies following suit. The result is a publishing system in which subscription prices no longer correspond to the quantity or quality of the work. The price per page charged by commercial publishers averaged six and seven times that of nonprofit society publishers across a number of disciplines in one study of journal subscriptions pricing [10]. This is not only about profits and surpluses, but about publishing practices that have been built up around these different structures over some time. If this current stratified economy had not been deemed unsustainable by those footing the bill, we might leave it at that. But some level of public accountability seems in order for a new digital age that holds such promise for universal access to knowledge, with one group of publishers exploring open access options with article processing fees in the area of US$3,000, while a new class of open-access journals takes advantage of open source software and new institutional capacities to publish peer-reviewed work without charge to either reader or author [11].

Which is only to say that the real battle for the future of science is not about NIH's provision of delayed access to author's drafts. It is about efforts to protect unsustainable revenue streams amid the capitalization of a public good to a degree that arguably undermines what is basic to the progress of science and useful arts. If the publishers' exclusive ownership of this body of work enables them to charge what they will, on their own terms, then what is at risk is the delicate-at-best balance between public interests in such learning and private investments in bringing it to the widest possible audience.

The NIH Public Access Policy stands as an expression of interest in righting the imbalance. It is a transition point, a step toward establishing a new economy of openness for the progress of science. No one has made that more clear than NIH director Elias A. Zerhouni in his testimony against the Fair Copyright bill [12]. Zerhouni spoke of how the digital revolution in the life sciences has led to far greater data and knowledge production, largely based on openly sharing genetic information and related sources through federally financed services, such as the National Center of Biotechnology Information (NCBI). And in terms of the contribution made by the NIH Public Access Policy, he reports that “400,000 users are accessing 700,000 articles every day” in PubMed Central [12].

Zerhouni spoke, as well, of public interest in this knowledge, pointing to how the majority of patients now turn up at the doctor's office having visited medical Web sites. This interest in access to knowledge is bound to spread to the other professions and their clients as well. Publishers, who could once count on no one giving a second thought, least of all researchers, to transferring to them the copyright for the entire research corpus, article by article, should have sufficient vision to see that a new civil rights issue is emerging over access to, as well as control over, such knowledge.

Now may be the time to step back from these time-consuming legislative skirmishes over who should determine rights of access to this knowledge. What makes far more sense at this point, as we move into a new publishing medium, is for journal publishers, scholarly societies, and research libraries to come to the table, to rethink cost structures, to collaborate on publishing platforms and systems, and to explore new ways of justifying, rationalizing, and allocating what is well over a US$7 billion investment in the circulation of knowledge (to use the figure for STM English-language publishing alone) [13]. What is needed are ways to maximize the progress of science and the useful arts as a function of their openness and public place.

You may find the prospects of such coordinated and cooperative approaches to advancing this public good hopelessly naive, even in this time of renewed hope and economic reconstruction. If so, then I can only advise constant, if not increased, vigilance on behalf of those with an interest in the openness of science. It will be a long road forward of strategic incremental measures, such as the NIH Public Access Policy, with carefully orchestrated counter-measures, even as a number of us within the academic community seek ways to extend this vision of public access to all that we do in the name of research and scholarship.
